# Extending the dosing interval of COVID-19 vaccination leads to higher rates of seroconversion in people living with HIV

**DOI:** 10.3389/fimmu.2023.1152695

**Published:** 2023-03-02

**Authors:** Yi Wang, Jianhua Li, Wenhui Zhang, Shourong Liu, Liangbin Miao, Zhaoyi Li, Ai Fu, Jianfeng Bao, Lili Huang, Liping Zheng, Er Li, Yanjun Zhang, Jianhua Yu

**Affiliations:** ^1^Department of Infection, Affiliated Hangzhou Xixi Hospital, Zhejiang University School of Medicine, Hangzhou, China; ^2^Institute of Hepatology and Epidemiology, Affiliated Hangzhou Xixi Hospital, Zhejiang University School of Medicine, Hangzhou, China; ^3^Institute of Microbiology, Zhejiang Provincial Center for Disease Control and Prevention (CDC), Hangzhou, China; ^4^Department of Nursing, Affiliated Hangzhou Xixi Hospital, Zhejiang University School of Medicine, Hangzhou, China; ^5^Medical Laboratory, Affiliated Hangzhou Xixi Hospital, Zhejiang University School of Medicine, Hangzhou, China

**Keywords:** inactivated COVID-19 vaccination, dosing interval, neutralizing antibody, seroconversion, people living with HIV

## Abstract

**Introduction:**

Vaccination against severe acute respiratory syndrome coronavirus 2 (SARS-CoV-2) infection is an effective way of protecting individuals from severe coronavirus disease 2019 (COVID-19). However, immune responses to vaccination vary considerably. This study dynamically assessed the neutralizing antibody (NAb) responses to the third dose of the inactivated COVID-19 vaccine administered to people living with human immunodeficiency virus (HIV; PLWH) with different inoculation intervals.

**Methods:**

A total of 171 participants were recruited: 63 PLWH were placed in cohort 1 (with 3-month interval between the second and third doses), while 95 PLWH were placed in cohort 2 (with 5-month interval between the second and third doses); 13 individuals were enrolled as healthy controls (HCs). And risk factors associated with seroconversion failure after vaccination were identified via Cox regression analysis.

**Results:**

At 6 months after the third vaccination, PLWH in cohort 2 had higher NAb levels (GMC: 64.59 vs 21.99, P < 0.0001) and seroconversion rate (68.42% vs 19.05%, P < 0.0001). A weaker neutralizing activity against the SARSCoV-2 Delta variant was observed (GMT: 3.38 and 3.63, P < 0.01) relative to the wildtype strain (GMT: 13.68 and 14.83) in both cohorts. None of the participants (including HCs or PLWH) could mount a NAb response against Omicron BA.5.2. In the risk model, independent risk factors for NAb seroconversion failure were the vaccination interval (hazed ration [HR]: 0.316, P < 0.001) and lymphocyte counts (HR: 0.409, P < 0.001). Additionally, PLWH who exhibited NAb seroconversion after vaccination had fewer initial COVID-19 symptoms when infected with Omicron.

**Discussion:**

This study demonstrated that the third vaccination elicited better NAb responses in PLWH, when a longer interval was used between vaccinations. Since post-vaccination seroconversion reduced the number of symptoms induced by Omicron, efforts to protect PLWH with risk factors for NAb seroconversion failure may be needed during future Omicron surges.

**Clinical trial registration:**

https://beta.clinicaltrials.gov/study/NCT05075070, identifier NCT05075070.

## 1 Introduction

Vaccination against severe acute respiratory syndrome coronavirus 2 (SARS-CoV-2) has been proved to efficiently decrease the likelihood of severe coronavirus disease 2019 (COVID-19). To optimize the outcomes of vaccination, considerable efforts have been devoted to the development of various vaccine strategies, such as trialing different inoculation intervals and COVID-19 vaccine types, to determine which combinations elicit the best immune responses. Administering a second dose of COVID-19 vaccines and prolonging the inoculation interval have improved vaccine immunogenicity not only in healthy controls (HC) (1) but also in patients undergoing hemodialysis (2), or those with autoimmune rheumatic disease (3) and cancer (4). People living with human immunodeficiency virus (HIV; PLWH) are a group of patients with impaired immunity. These individuals were therefore regarded as a priority population for COVID-19 vaccination and were highly recommended to receive the third vaccine dose. Thus, the potential value of extending the inoculation interval between the second and third doses of the COVID-19 vaccine deserved further attention.

Although existing data demonstrate that COVID-19 vaccines could elicit neutralizing antibody (NAb) responses in PLWH, NAb could not fully account for the effectiveness of the vaccines. Furthermore, since SARS-CoV-2 can rapidly mutate and most COVID-19 vaccines were designed against the wild-type (WT) Wuhan strain, it is important to determine the NAb activity against SARS-CoV-2 variants. To date, studies performed in Canada (5), Spain (6), and China (7) suggested that two doses of COVID-19 vaccine induced adequate levels of NAb against the WT SARS-CoV-2 strain in PLWH. Moreover, a South African study by Khan et al. showed that inoculating PLWH with a single dose of the Ad26.CoV2.S vaccine induced a considerable NAb response against the Delta variant (8). Very recently, inoculation of PLWH with a third dose of an mRNA COVID-19 vaccine was shown to elicit a robust NAb response against the Omicron BA.1 variant (9, 10). The emergence of an increasing number of Omicron sub-lineages, which have a higher propensity for immune evasion compared with the Omicron variant BA.1, has been reported (11, 12). However, only a single cross-sectional study, has evaluated the NAb responses of PLWH against Omicron BA.4/5 variants (13). In addition, there is lack of evidence of NAb responses against Omicron sub-lineages in PLWH before and after the third vaccination.

This study aimed to reveal the influence of extending the inoculation interval on the humoral immunity induced by the third dose of the inactivated COVID-19 vaccine in PLWH with a CD4 count < 500 cells/μL. Of note, this work is a continuation of our previous research, which forms part of a long-term follow-up program (under review). In the present study, we measured the dynamic NAb responses of PLWH to different SARS-CoV-2 strains, including the Omicron BA.5.2 variant, which is one of the most prevalent strains in China. Moreover, potential factors linked to NAb seroconversion were identified. Collectively, our results will provide additional guidance for the COVID-19 vaccination of PLWH.

## 2 Materials and methods

### 2.1 Study population and design

**Figure 1:**
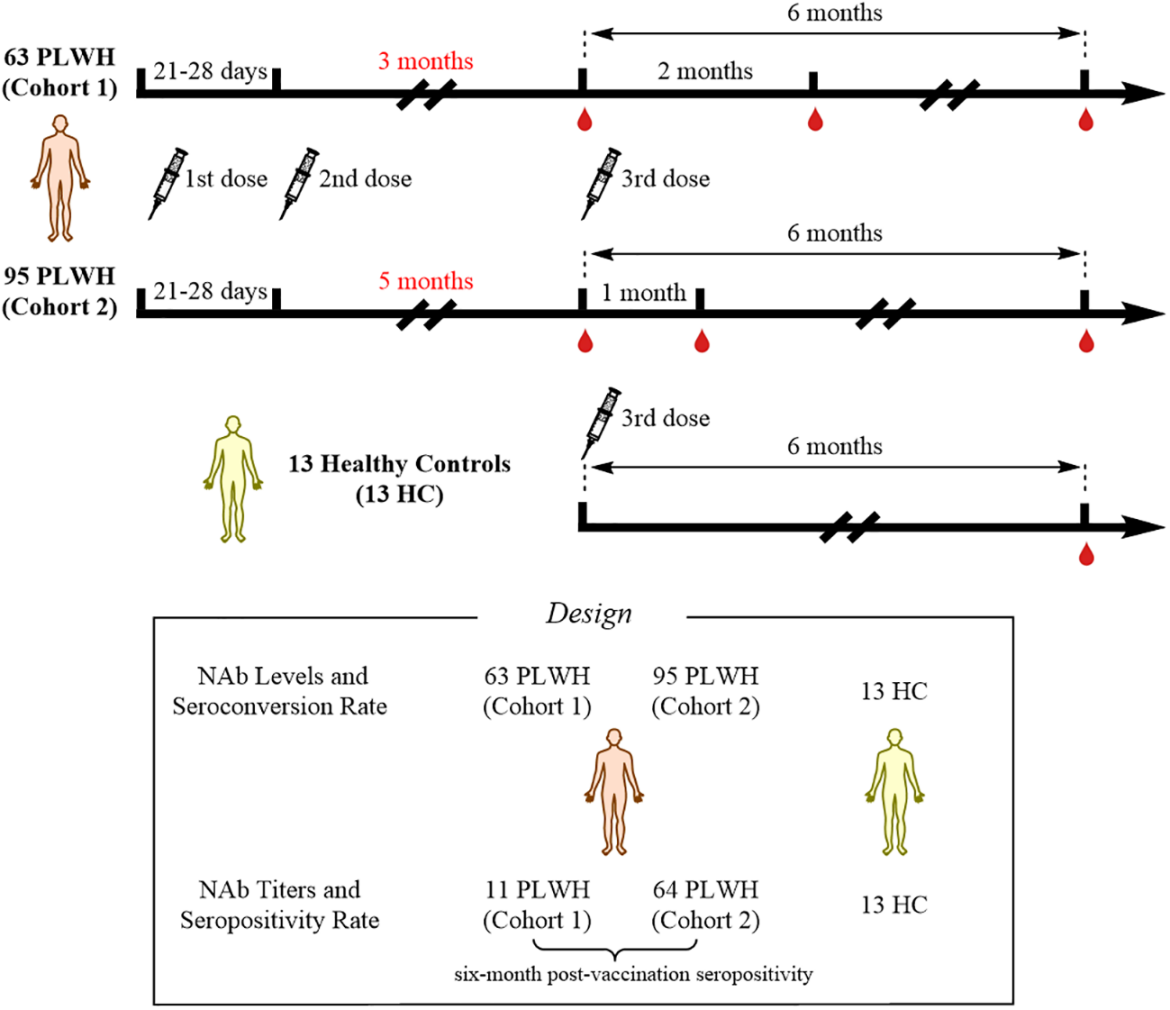
Study profile for the vaccination schedule and follow-up.

This was an observational study that collected data from PLWH vaccinated with inactivated COVID-19 vaccines (called BBIBP-CorV, Beijing Institute of Biological Products) at Hangzhou Xixi Hospital. At enrollment, all PLWH received standardized antiretroviral therapy (ART), and had viral loads less than 50 copies/mL and CD4^+^ T-cell counts less than 500 cells/μL. All PLWH were SARS-CoV-2-negative throughout the study period. A total of 158 PLWH, who had received two doses of the inactivated COVID-19 vaccine, were enrolled in this study. Based on their vaccination willingness, the 158 individuals were divided into two cohorts according to the interval between the second and the third doses of vaccine: 1) cohort 1, interval of 3 months; and 2) cohort 2, interval of 5 months. The original plan was to collect peripheral blood samples at three time points to evaluate the NAb responses: 1) prior to the third vaccination; 2) 1 month after the third vaccination; and 3) 6 months after the third vaccination. However, due to the COVID-19 pandemic and quarantine policies in China, the second time-point for collecting peripheral blood samples from cohort 1 was changed to 2 months after the third dose, while cohort 2 was sampled according to the original plan. In addition, 13 HCs were recruited; data from these individuals were collected 6 months after the third dose of vaccination. The study flowchart is shown in **Figure 1**. This study was reviewed and approved by the Clinical Research Ethics Committee of the Hangzhou Xixi Hospital (202109131211000115379) and was performed in accordance with the Declaration of Helsinki. This study was registered on *clinicaltrials.gov* (NCT05075070). The infection status and initial symptoms of all PLWH were collected using a questionnaire during additional follow-up.

### 2.2 SARS-CoV-2 NAb detection assay

The SARS-CoV-2 NAb assay (SHENZHEN YHLO BIOTECH CO., LTD, Shenzhen, China) is a paramagnetic particle chemiluminescent immunoassay designed for the qualitative detection of SARS-CoV-2 NAb in human serum samples using the automated iFlash immunoassay system (14). This assay is mainly used for the auxiliary evaluation of the efficacy of inactivated COVID-19 vaccines. The manufacturer has determined a cut-off value of 10 arbitrary units per milliliter (AU/mL) for NAb levels. Since the maximum measurable result is 800 AU/mL, anti-SARS-CoV-2 NAb levels above 800 AU/mL were assigned as a value of 800. Seroconversion was defined as a change in the status of NAb levels from negative to positive based on a 4-fold increase over baseline (15). At the same time, according to Ka-Shing Cheung et al. study, we also set a assay threshold of 15 AU/mL as the NAb seroconversion cutoff in the our study (16). The geometric mean concentration (GMC) of NAb was calculated with 95% confidence intervals (CI), according to WHO international standards (17).

### 2.3 Serum neutralization of live SARS-CoV-2 strains

Viral neutralization assays were performed in a BSL-3 laboratory. The sera from participants were heat-inactivated at 56°C for 30 min prior to use. The working stocks of live SARS-CoV-2 strains (the WT strain and the Delta B.1.617.2 and Omicron BA.5.2 variants) were obtained from sputum samples and propagated by infection of Vero E6 cells, as previously described (18). The serum neutralizing activity was measured using the micro-neutralization assay based on live SARS-CoV-2 virus, as previously described (19). Virus without serum and untreated E6 cells were used as positive and negative controls, respectively. Microplates were observed under the microscope for the presence of virus-induced cytopathic effects (CPE) on the cell monolayer on days 2 to 3. Serum neutralization titers were calculated on day 3 as the reciprocal of the serum dilution that resulted in a 100% reduction in the CPE (20). We calculated the geometric mean titer (GMT) of NAb with 95% CIs, according to the WHO international standard (17). A seropositivity threshold was defined as a GMT over 1:4 (21, 22). In addition, NAb titers < 4 were assigned as a titer value of 2 (23).

### 2.4 Outcomes of interest

The primary outcome was NAb seroconversion within 6 months after the third dose of inactivated COVID-19 vaccine in PLWH. The NAb level prior to the third vaccination was set as the baseline for this study. After the third vaccination, PLWH with a ≥ 4-fold increase in NAb levels relative to the baseline were defined as those who underwent NAb seroconversion, and were called seroconverters. PLWH who did not meet these standards were regarded as non-seroconverters. The secondary outcomes were the incidence of virologically confirmed SARS-CoV-2 infection and the number of initial symptoms post-infection among the vaccinated PLWH.

### 2.5 Statistical analysis

All statistical analysis was performed using the R statistical software version 4.1.3 (R Foundation for Statistical Computing, Vienna, Austria) and IBM SPSS Statistic version 25.0 (IBM, Armonk, NY, USA). We assessed the statistical differences in NAb levels and titers elicited by different SARS-CoV-2 strains between the two cohorts using the Mann-Whitney *U*-test (for unpaired data) or the Wilcoxon rank-sum test (for paired data). Normally distributed continuous variables were presented as means with standard deviations (SD). The median (M) with interquartile range (IQR, 1^st^ quartile–3^rd^ quartile) were used to describe variables. For categorical variables, we reported the numbers and percentages of patients in each category. Proportions were compared using the Pearson’s chi-squared test. All graphs were generated using GraphPad Prism version 8.0.2 (263) (GraphPad Software, San Diego, CA, USA).

A multivariable Cox regression model was applied to determine covariate association with NAb seroconversion. A hazard ratio (HR) greater or less than 1 was interpreted as an increased or decreased association with NAb seroconversion, respectively. We used the “Survival” and “Survminer” packages in R to analyze the NAb seroconversion data from PLWH. The predictive accuracy of the risk model was assessed using the receiver operating characteristic (ROC) curve, and the area under ROC curve (AUC) was plotted by the MedCalc software package version 18.2.1 (MedCalc Software, Ostend, Belgium) using sensitivity and specificity values (24). Kaplan-Meier curves were drawn for PLWH with or without NAb seroconversion and compared using log-rank tests. All reported levels of statistical significance were two-sided, and *P-*values < 0.05 were considered as a measure of statistical significance.

## 3 Results

### 3.1 Baseline characteristics of PLWH

In total, 158 PLWH received the third dose of inactivated COVID-19 vaccine, of which 63 PLWH were in cohort 1 (with a 3-month interval between the second and third doses) and 95 PLWH were in cohort 2 (with a 5-month interval between the second and third doses). The baseline characteristics mainly included age, sex, HIV transmission route, marital status, education, body mass index (BMI), time at initiation of ART, and CD4^+^ T-cell counts. There were no obvious differences in these characteristics between the two cohorts at baseline (**Table 1**).

**Table 1:** The comparison of baseline characteristics of participants between two cohorts.

Characteristics	Cohort 1 (n = 63)	Cohort 2 (n = 95)	*P* value
Age (years)			
<30	14 (22.22)	27 (28.42)	0.607
30-40	22 (34.92)	39 (41.05)	
41-50	12 (19.05)	12 (12.63)	
≥50	15 (23.81)	17 (17.90)	
Sex			
Male	61 (96.83)	92 (96.84)	1.000
Female	2 (3.17)	3 (3.16)	
Sexual transmission route			
Homosexual/bisexual	47 (74.60)	76 (80.00)	0.782
Heterosexual	15 (23.81)	18 (18.95)	
Others	1 (1.59)	1 (1.05)	
Marital status			
Married	21 (33.33)	28 (29.47)	0.876
Unmarried	37 (58.73)	59 (62.11)	
Divorced/widowed	5 (7.94)	8 (8.42)	
Education			
High school or lower	19 (30.16)	29 (30.53)	0.620
Junior college	18 (28.57)	21 (22.11)	
College or higher	26 (41.27)	45 (47.37)	
BMI	22.33 ± 3.36	21.80 ± 3.97	0.380
Time at initiation of treatment/years			
<2	13 (20.63)	17 (17.89)	0.224
2-5	15 (23.81)	35 (36.84)	
≥5	35 (55.56)	43 (45.26)	
CD4^+^ T-cell counts (cells/μL)			
<200	19 (30.16)	25 (26.32)	0.690
200-350	23 (36.51)	40 (42.11)	
350-500	21 (33.33)	30 (31.57)	

BMI, body mass index.

### 3.2 The NAb response to the third dose of vaccination in cohort 1

**Figure 2:**
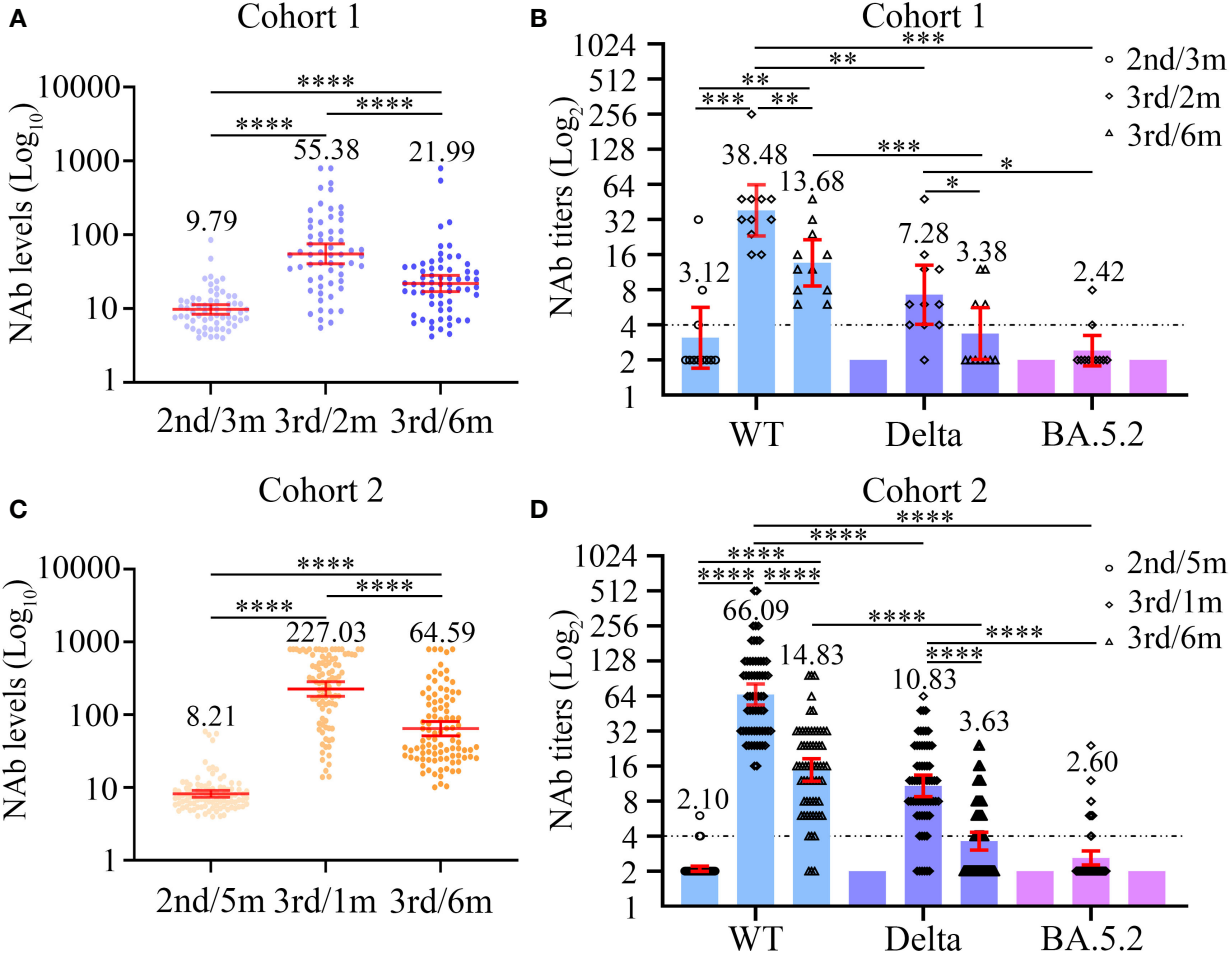
Dynamic changes in neutralizing antibody levels **(A, C)** and titers **(B, D)** to wild-type virus and the Delta (B.1.617.2) and Omicron (BA.5.2) variants before and after the third dose of COVID-19 vaccines among two cohorts. **(A, B)** The neutralizing antibody levels **(A)** and titers **(B)** at three different time points in cohort 1. **(C, D)** The neutralizing antibody levels **(C)** and titers **(D)** at three different time points in cohort 2. Wilcoxon matched-pairs signed-rank test with two-tailed p-value was used for comparison between groups. *P < 0.05, **P < 0.01, ***P < 0.001, ****P < 0.0001.

**Figure 3:**
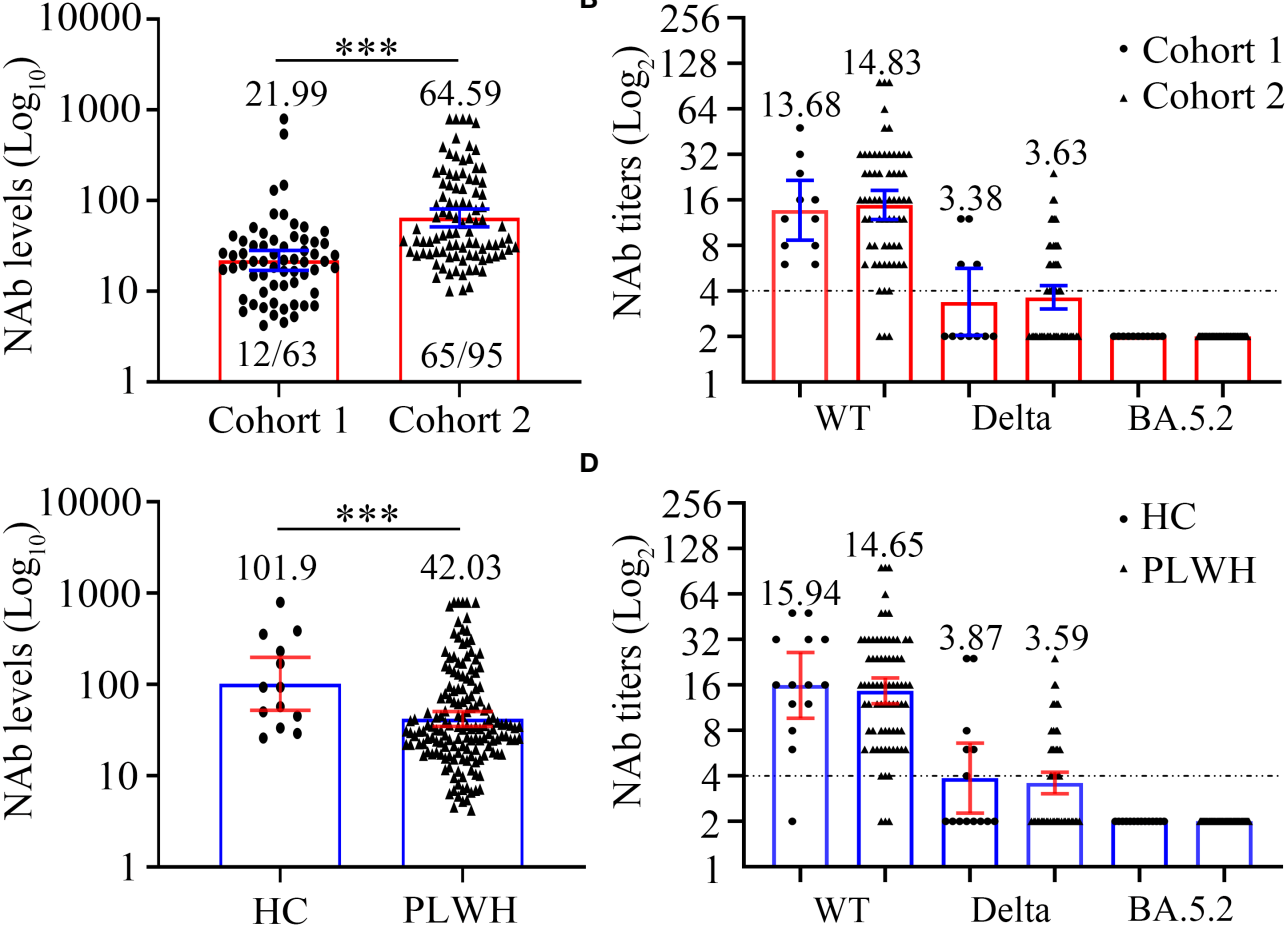
Comparison of SARS-CoV-2 neutralizing antibody levels **(A, C)** and titers **(B, D)** to wild-type virus and the Delta (B.1.617.2) and Omicron (BA.5.2) variants between two cohorts or PLWH and healthy controls at 6 months after the third vaccination. **(A, B)** The neutralizing antibody levels **(A)** and titers **(B)** of cohort 1 and cohort 2. **(C, D)** The neutralizing antibody levels **(C)** and titers **(D)** of PLWH and healthy controls. Mann-Whitney U test with two-tailed p-value was used for comparison between groups. ***P < 0.001.

NAb levels and titers were characterized at three different time points in cohort 1. Compared with the baseline NAb levels (GMC = 9.79), the significant increase in the magnitude of the NAb response was observed at 2 months after the third dose of inactivated COVID-19 vaccine among the 63 PLWH of cohort 1 (GMC: 9.79 vs 55.38, *P* < 0.0001, **Figure 2A**; **Table S1**). At the 6 months post-vaccination time point, the GMC of NAb was lower that at the second time point (GMC: 55.38 vs 21.99; *P* < 0.0001, **Table S1**), but was still significantly higher than baseline (GMC: 9.79 vs 21.99; *P* < 0.0001, **Table S1**). Furthermore, a total of 12 PLWH in cohort 1 experienced NAb seroconversion (**Figure 3A**).

In addition, we assessed the NAb seroconversion rate and levels at 6-month post-third vaccination in PLWH with a CD4 count < 200 cells/µL (PLWH < 200) or ≥ 200 cells/µL (PLWH ≥ 200) subgroups to identify potential differences. The baseline characteristics of PLWH < 200 and PLWH ≥ 200 subgroups are showed in **Table S2**. As showed in **Table 2**, the NAb seroconversion rate (0.00% vs 27.27%) and levels (11.60 vs 24.95 AU/mL) in PLWH < 200 subgroup were significantly lower than those in PLWH ≥ 200 subgroup (Both *P* < 0.05, **Table 2**).

**Table 2:** The comparison of NAb seroconversion rate and levels of participants between two subgroups in cohort 1.

Varibles	PLWH < 200 (n = 19)	PLWH ≥ 200 (n = 44)	*P* value
Seroconversion rate			
No (n,%)	19 (100)	32 (72.73)	0.029
Yes (n,%)	0 (0.00)	12 (27.27)	
NAb level	11.60 (6.40,25.80)	24.95 (16.60,39.45)	0.007

PLWH < 200: PLWH with a CD4 count < 200 cells/µL; PLWH ≥ 200: PLWH with a CD4 count ≥ 200 cells/µL.

Next, we carried out live SARS-CoV-2 neutralization test in PLWH seroconverters. Except for one individual, who supplied insufficient blood sample volume, the remaining 11 PLWH were included in the next evaluation of neutralizing activity against different SARS-CoV-2 strains. Before the third vaccination, only three PLWH displayed the ability to neutralize live SARS-CoV-2 WT, and none of the 11 PLWH exhibited neutralization activity against live SARS-CoV-2 variants (**Figure 2B**; **Table S1**). Among the 11 PLWH, the detectable rate of neutralization activity was 11 of 11 (100%), 10 of 11 (90.91%), and 2 of 11 (18.18%); the GMTs were 38.48, 7.28, and 2.42 to WT, Delta, and Omicron strains 2 months after the third vaccination, respectively (**Figure 2B**; **Table S1**). The serum samples taken at the 6-month time point had lower neutralization activity to the three SARS-CoV-2 strains. At 6 months, the NAb GMTs against WT and Delta strains significantly decreased to 13.68 and 3.38, respectively (**Figure 2B**; **Table S1**), while none of the samples could neutralize the Omicron variant (**Figure 2B**; **Table S1**).

### 3.3 The NAb response to the third dose of vaccination in cohort 2

Among the 95 PLWH in cohort 2, the NAb responses to the third dose of inactivated COVID-19 vaccine were significantly elevated compared with those to the second dose, both at the 1-month and 6-month time points (both *P* < 0.0001, **Figure 2C**; **Table S3**). However, the NAb GMCs significantly dropped between the 1-month and 6-month time points (227.03 vs 64.59; *P* < 0.0001, **Figure 2C**; **Table S3**). In addition, 6 months after the third vaccination, 65/95 (68.42%) PLWH experienced NAb seroconversion (**Figure 3A**).

Then, the NAb seroconversion rate and levels at 6-month post-third vaccination were assessed in PLWH < 200 or PLWH ≥ 200 subgroups to identify potential differences. The baseline characteristics of PLWH < 200 and PLWH ≥ 200 subgroups are showed in **Table S4**. After subgroups analysis, there were no significant differences in the NAb seroconversion rate (76.00% vs 65.71%) and levels (44.80 vs 46.40 AU/mL) between PLWH < 200 and PLWH ≥ 200 subgroups (Both *P* > 0.05, **Table 3**).

**Table 3:** The comparison of NAb seroconversion rate and levels of participants between two subgroups in cohort 2.

Varibles	PLWH < 200 (n = 25)	PLWH ≥ 200 (n = 70)	*P* value
Seroconversion rate			
No (n,%)	6 (24.00)	24 (34.29)	0.322
Yes (n,%)	19 (76.00)	46 (65.71)	
NAb level	44.80 (26.25,191.50)	46.40 (26.75,144.50)	0.857

PLWH < 200: PLWH with a CD4 count < 200 cells/µL; PLWH ≥ 200: PLWH with a CD4 count ≥ 200 cells/µL.

Except for one PLWH, who supplied insufficient blood sample volumes, the remaining 64 PLWH seroconverters were selected to participate in the live SARS-CoV-2 neutralization test (**Figure 2D**; **Table S3**). At baseline, the sera from most PLWH could hardly neutralize live SARS-CoV-2 WT and the two variants. At 1 month post-vaccination, however, the sera of all PLWH (64/64, 100%) neutralized SARS-CoV-2 WT. Protection against the Delta variant was also built up in most PLWH (58/64, 90.63%). Nevertheless, the sera from only 14 PLWH (14/64, 21.88%) displayed neutralizing activity against the Omicron BA.5.2 variant. The NAb GMTs against the SARS-CoV-2 WT, Delta, and Omicron strains were 66.09, 10.83 and 2.60, respectively. An apparent drop in NAb GMTs was observed over time. At the 6-month time point, the NAb GMTs against SARS-CoV-2 WT and Delta strains dramatically decreased to 14.83 and 3.63, respectively, and none of the sera from cohort 2 participants were able to neutralize the Omicron BA.5.2 variant.

Besides, we further observed the differences between PLWH < 200 and PLWH ≥ 200 subgroups on the neutralizing activity against different SARS-CoV-2 strains among PLWH seroconverters in cohort 2. We found no evidence of a different titer of NAbs neutralization against SARS-CoV-2 WT, Delta, and Omicron strains between PLWH < 200 and PLWH ≥ 200 subgroups at one and six-month post-vaccination (**Table S5**).

### 3.4 Differences between the NAb responses of the two cohorts 6 months after the third vaccination

Firstly, we compared the NAb levels and seroconversion rate in the two cohorts 6 months after the third vaccination. As shown in **Figure 3A** and **Table 4**, the PLWH in cohort 2 (with a longer vaccination interval) had markedly higher seroconversion rate than those in cohort 1 (68.42% vs 19.05%, *P* < 0.0001). This trend was also observed with respect to the concentrations of SARS-CoV-2-specific NAbs in cohort 2 vs cohort 1 (GMC: 64.59 vs 21.99, *P* < 0.0001). Notably, both PLWH < 200 and PLWH ≥ 200 subgroups, the NAb levels and seroconversion rate in cohort 2 were significantly higher than those in cohort 1 (*P* < 0.0001, **Table 4**).

**Table 4:** The differences on the neutralizing antibody levels between two cohorts at 6 months post-3rd vaccination.

Neutralizing Antibody	Cohort 1 (n = 63)	Cohort 2 (n = 95)	*P* value
Total seroconversion rate (n,%)	12/63 (19.05)	65/95 (68.42)	< 0.0001
Total GMC (95%CI)	21.99 (17.05,28.34)	64.59 (51.34,81.26)	< 0.0001
PLWH < 200 seroconversion rate (n,%)	0/19 (0.00)	19/25 (76.00)	< 0.0001
PLWH < 200 GMC (95%CI)	12.99 (8.87,19.01)	67.74 (41.38,110.90)	< 0.0001
PLWH ≥ 200 seroconversion rate (n,%)	12/44 (27.27)	46/70 (65.71)	< 0.0001
PLWH ≥ 200 GMC (95%CI)	27.60 (20.30,37.60)	63.50 (48.73,82.75)	< 0.0001

GMC, geometric mean concentration; CI, confidence interval; PLWH < 200: PLWH with a CD4 count < 200 cells/µL; PLWH ≥ 200: PLWH with a CD4 count ≥ 200 cells/µL.

In addition to the levels of NAb, we also compared the neutralizing activity against different SARS-CoV-2 strains (**Figure 3B**; **Table S6**). Interestingly, regardless of the targeted SARS-CoV-2 strain (WT, Delta, or Omicron), the neutralizing activities were comparable between the PLWH seroconverters in the two cohorts. Specifically, the GMTs against the WT strain in cohort 1 and cohort 2 were 13.68 and 14.83, respectively, and a sharp reduction in the GMTs against the Delta strain was observed (GMT: 3.38 and 3.63, respectively, for cohorts 1 and 2). Furthermore, the data revealed that neither vaccination regiment was able to elicit NAbs against Omicron BA.5.2.

### 3.5 Differences between the NAb responses of PLWH and healthy controls 6 months after the third vaccination

The baseline characteristics of HC and PLWH, including age, sex, and BMI, are showed in **Table S7**. In the initial analysis, we compared the NAb levels 6 months after the third vaccination in 158 PLWH and 13 HCs. As shown in **Figure 3C** and **Table S8**, PLWH had significantly lower NAb levels than controls (GMC: 42.03 vs 101.90, *P* = 0.0056). We then examined NAb titers in 75 PLWH seroconverters and 13 HCs with high NAb levels. The GMTs of serum NAb against WT strain were 15.94 and 14.65, while those against the Delta strain were 3.87 and 3.59 for the PLWH seroconverters and HCs, respectively (**Figure 3D**; **Table S8**). At 6 months post-vaccination, the sera of both PLWH and HCs were unable to neutralize the Omicron variant (**Figure 3D**; **Table S8**).

### 3.6 Factors associated with NAb seroconversion in PLWH

We next focused on identifying factors associated with NAb seroconversion. We used the univariate Cox regression analysis to calculate the HRs for 22 factors (**Table S9**). Our results showed that six variables, including age (HR = 2.057; 95%CI, 1.233–3.433; *P* = 0.006), sex (HR = 2.542; 95%CI, 1.022–6.325; *P* = 0.045), vaccination interval (HR = 0.336; 95%CI, 0.212–0.531; *P* < 0.0001), education (HR = 0.467; 95%CI, 0.282–0.775; *P* = 0.003), lymphocyte counts (HR = 0.593; 95%CI, 0.395–0.889; *P* = 0.011), and neutrophil-to-lymphocyte ratios (NLRs) (HR = 1.289; 95%CI, 1.069–1.553; *P* = 0.008) were independent significant predictors for NAb seroconversion (**Table S9**).

We then calculated the cut-off values of two continuous variables, which were significantly associated with the outcome of NAb seroconversion. The optimal cut-off value of lymphocyte counts was ≤ 1.59 cells/μL, with a sensitivity and specificity of 43.21% and 77.92%, respectively. The NLRs had 45.68% sensitivity and 64.94% specificity at an optimal cut-off value of > 1.96 (**Table S10**).

**Figure 4:**
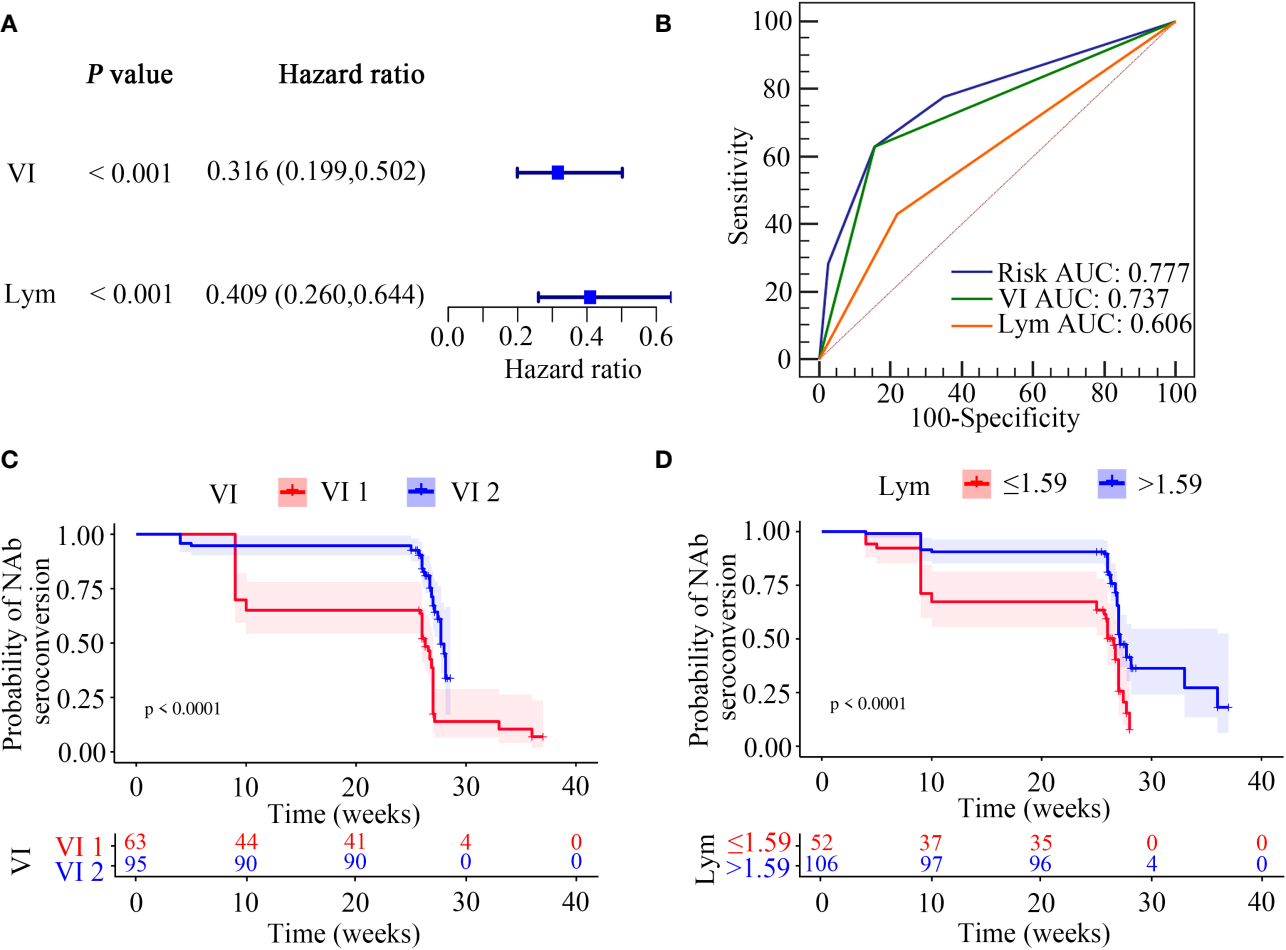
Screening of NAb seroconversion related factors. **(A)** Multivariate cox regression analysis (*P* < 0.05). **(B)** ROC demonstrated the predictive accuracy of the risk model was superior to other two clinical variables. **(C, D)** Kaplan-Meier survival function plot showed the effect of vaccination interval and lymphocyte counts on the outcome of NAb seroconversion.

We next used the multivariable Cox regression analysis to screen out two variables, namely the vaccination interval (HR = 0.328; 95%CI, 0.204–0.528; *P* < 0.0001) and lymphocyte counts (HR = 0.497; 95%CI, 0.307–0.805; *P* = 0.004), which were significantly associated with NAb seroconversion (**Table 5**). The results were presented in the form of forest maps (**Figure 4A**). We then used ROC analysis to assess the ability of these two independent variables to predict the outcome of NAb seroconversion among PLWH. In the ROC analysis chart, the AUC values for the vaccination interval and lymphocyte counts were 0.737 (95%CI, 0.661–0.804; *P* < 0.0001) and 0.606 (95%CI, 0.525–0.682; *P* = 0.0038), respectively (**Figure 4B**; **Table S11**). We constructed a risk model with an AUC of 0.777 (95%CI, 0.704–0.840; *P* < 0.0001), based on the vaccination interval and lymphocyte counts (**Figure 4B**; **Table S11**). PLWH with a longer interval between doses (5 months) were more likely to experience NAb seroconversion than PLWH with a shorter interval (3 months) between doses (**Figure 4C**, *P* < 0.0001). Lower lymphocyte counts were associated with a significant decline in NAb seroconversion (**Figure 4D**, *P* < 0.0001).

**Table 5:** Multivariate Cox regression analysis was used to analyze the factors of the outcome of NAb seroconversion among PLWH after the third vaccination.

Variables	*β* value	SE	Wald χ^2^	*P* value	HR (95%CI)
Age (years)					
≥50 vs <50	0.417	0.329	1.609	0.205	1.517 (0.797,2.889)
Sex					
female vs male	0.781	0.497	2.476	0.116	2.185 (0.825,5.781)
Vaccination interval					
2 vs 1	-1.113	0.242	21.156	< 0.0001	0.328 (0.204,0.528)
Education			2.428	0.297	
2 vs 1	-0.322	0.327	0.969	0.325	0.725 (0.382,1.375)
3 vs 1	-0.475	0.306	2.408	0.121	0.622 (0.34,1.133)
Lymphocytes (cells/μL)					
>1.59 vs ≤1.59	-0.698	0.246	8.079	0.004	0.497 (0.307,0.805)
NLRs					
>1.96 vs ≤1.96	0.282	0.241	1.366	0.242	1.326 (0.826,2.129)

SE, standard error; HR, hazard ratio; NLRs, neutrophil/lymphocyte ratios.

**Figure 5:**
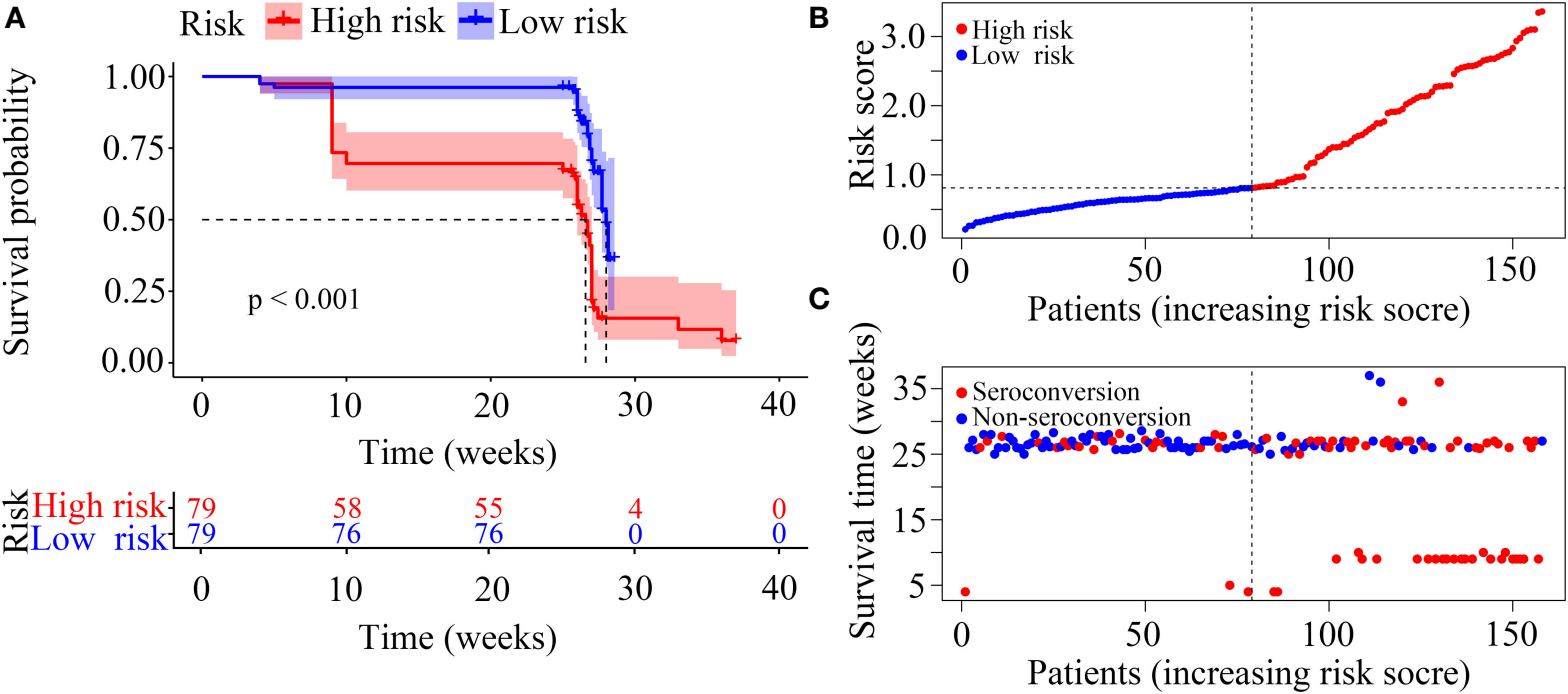
Construction of the risk score model with respect to PLWH without NAb seroconversion. **(A)** Kaplan-Meier curves of the overall survival of the two risk groups of PLWH with NAb seroconversion. **(B, C)** Risk score curves and scatter plots of the risk for PLWH without NAb seroconversion.

In our risk model, the PLWH were divided into two groups according to risk (high vs low), and the grouping criterion was the median of the risk score. The subsequent survival analysis revealed that the status of NAb seroconversion between the two groups was significantly different (**Figure 5A**, *P* < 0.0001). The risk curve shows the relationship between NAb seroconversion and the risk of PLWH. **Figure 5B** shows the risk values of PLWH in the two groups. PLWH in the low-risk group had significantly higher NAb levels and seroconversion rate than those in the high-risk group (**Figure 5C**).

### 3.7 PLWH seroconverters have fewer SARS-CoV-2-induced symptoms

To prove the clinical relevance of the model, we recently conducted an additional follow-up to investigate the incidence rate and initial symptoms of SARS-CoV-2 (Omicron variant) breakthrough infection in the 158 vaccinated PLWH. Within 1 year after the third vaccination, a total of 108 PLWH tested positive for SARS-CoV-2 RNA and 24 PLWH had negative results. SARS-CoV-2 RNA test results could not be obtained for the remaining 26 PLWH, who were subsequently excluded form the analysis. Among the 132 PLWH, 60 experienced NAb seroconversion and 72 did not. As shown in **Table S12**, the rate of infection with SARS-CoV-2 Omicron variant was slightly higher in non-seroconverters compared to seroconverters; however, this did not reach statistical significance (80.00% vs 83.33%, *P* > 0.05).

Further analysis was focused on the 108 PLWH infected with the SARS-CoV-2 Omicron variant, including 48 seroconverters and 60 non-seroconverters. The baseline characteristics between the SARS-CoV-2-positive seroconverters and non-seroconverters were comparable (**Table S13**). Interestingly, the number of initial symptoms induced by the SARS-CoV-2 Omicron variant was significantly lower in seroconverters than in non-seroconverters (**Table 6**, *P* = 0.027); **Table S14** lists the ten most common COVID-19-related symptoms experience by the 108 PLWH, including fever, cough, sore throat, muscle aches, nasal obstruction, runny nose, headache, loss of appetite, loss of taste and smell, and abdominal pain and diarrhea.

**Table 6:** The association between NAb seroconversion and the number of symptoms induced by SARS-CoV-2 Omicron variant infection in PLWH.

Number of Symptoms	Seroconverters (n = 48)	Non-seroconverters (n = 60)	*P* value
≤ 2 (n, %)	15 (31.25)	8 (13.33)	0.027
> 2 (n, %)	33 (68.75)	52 (86.67)	

## 4 Discussion

There is insufficient evidence on the efficacy of three consecutive doses of inactivated COVID-19 vaccine against SARS-CoV-2 variants, especially in PLWH. This study dynamically evaluated the NAb response (NAb levels and titers at 6 months post-vaccination) in two PLWH cohorts with different vaccination intervals. We then built a risk model to predict the outcome of NAb seroconversion after vaccination and found that the risk of NAb seroconversion failure among PLWH was inversely proportional to lymphocyte counts and a vaccination interval. Once the COVID-19 restriction were eased in China, we were able to perform an additional follow-up, whereby we compare the frequency of SARS-CoV-2 infections and the number of COVID-19-related symptoms in the PLWH seroconverters and non-seroconverts after the third vaccination.

A substantial increase in NAb levels was observed in both cohorts after the third vaccination, which declined rapidly with time. The dynamics of NAb levels after the third dose of inactivated COVID-19 vaccine are in line with those elicited by other types of COVID-19 vaccines in immunocompromised populations worldwide (25, 26). 6 months after the third vaccination, nearly half of the PLWH (81/158) in our study failed to undergo NAb seroconversion. An interesting study showed that initially poor vaccine responders could generate 5.4-fold higher NAb levels following an additional vaccine dose (27). The above data imply that PLWH with a poor immune response to COVID-19 vaccination (28) should receive a booster vaccine dose.

Since the escape of SARS-CoV-2 variants from NAbs has been widely reported (29), it is necessary to evaluate the NAb responses against the Delta and Omicron variants. In the short-term follow-up period after the third vaccination, the serum samples of PLWH seroconverters from both two cohorts exhibited high neutralization activities against the WT and Delta strains, but not the Omicron variant. While the NAb levels of most PLWH were sufficiently high after the third dose of vaccination, only a minority PLWH (two participants in cohort 1 and 14 participants in cohort 2) developed effective antibody titers against the Omicron BA.5.2 variant over short-term follow-up. The main reason for this is likely that the current COVID-19 vaccine was designed to target the WT SARS-CoV-2 strain (30) and the Omicron variant differs more from the WT than the Delta variant (31). The results of a cross-sectional study showed that NAbs elicited in PLWH by the third vaccination were only mildly effective at inhibiting the replication of the BA.4/5 variant (13). By contrast, Vergori et al. observed that the third dose of the COVID-19 mRNA vaccine could effectively prevent infection of PLWH with SARS-CoV-2 strains (Wuhan-D614G and Omicron BA.1) within 2 weeks of vaccination (9). The weak neutralizing ability of NAbs against Omicron BA.5.2 observed in our study could be attributed to a longer interval for blood collection or the difference between COVID-19 vaccines. Another possible explanation is the differences in the immunogenicity of the BA.5.2 and BA.1 Omicron variants (10, 13). At 6 months after the third vaccination, the efficacy of the NAb response against the SARS-CoV-2 strains was inadequate in both cohorts; this was especially true for the Omicron BA.5.2 variant. A similar phenomenon was reported by Lapointe et al. and Zhan et al. (10, 13). Given that none of the PLWH exhibited neutralization activity against Omicron BA.5.2, we analyzed the sera from HCs to see whether the persistent immune dysregulation and chronic inflammatory status of PLWH (32) reduced their ability to mount a protective NAb response against Omicron (30). We found that HCs had significantly higher NAb levels than PLWH after vaccination, as previously reported (7, 13, 33). Interestingly, despite of higher NAb levels, the anti-Omicron-BA.5.2 NAb titers were undetectable in both PLWH and HCs. Indeed, the SARS-CoV-2 Omicron sub-variants are highly resistant to neutralization by NAbs induced by the COVID-19 vaccine, even in healthy participants (34). This could be attributed to the fact the Omicron variant undergoes large-scale mutations in the RBD region of the spike protein, which enable it to effectively evade the immune response (35, 36). This could also account for the occurrence of breakthrough infections involving the Omicron variant. Thus, the development of more effective Omicron-based vaccines is urgently needed.

Previous studies have indicated that a longer interval between the first and second doses of COVID-19 adenovirus vector and mRNA vaccine induced more immunogenic responses (37, 38). As a complement, we evaluated NAb responses 6 months after the third dose of inactivated COVID‐19 vaccines in PLWH with different inoculation intervals. In accordance with the previous studies, we found that a longer interval between the second and third doses of COVID-19 vaccine was associated with markedly higher NAb levels (GMC: 64.59 vs 21.99 AU/mL) and seroconversion rates. The explanation could be extending the dosing interval may increase the B cells selection stringency and boost the formation of memory B cells, those would exhibit stronger antibody responses to the next-dose COVID-19 vaccine (39). Another study also pointed that a longer dosing interval may allow antibodies to mature for longer, and lead to enhanced immunogenicity and efficacy (40). These evidences supported the antibody responses in the longer interval group was better than those in the shorter interval group. The above data confirmed that an appropriate length of interval between COVID-19 vaccine doses should be part of an effective vaccination protocol (41). An optimal inoculation interval would reduce inoculation frequency and therefore limit costs, which is particularly important in the resource-poor areas with a shortage of vaccine supply.

Previous studies have shown that lower CD4^+^ T cell count has been linked to lower serological responses among PLWH (42). Consistent with several prior studies (9, 43, 44), our study also indicated that PLWH < 200 subgroup in cohort 1 (3-month interval) showed a weaker humoral immune response to inactivated COVID-19 vaccination, comparing to PLWH CD4 ≥ 200 subgroup (*P* < 0.05). Interestingly, the gap in NAb levels and seroconversion rates disappeared as extending the dosing interval to 5-month in PLWH < 200 and PLWH ≥ 200 subgroups. The above data implied the appropriate dosing interval played a crucial role in PLWH with low CD4 count (< 200 cells/μL).

Furthermore, our study showed that an appropriate vaccination interval was as one predictor of the outcome of NAb seroconversion. Additionally, a decrease in the number of lymphocytes was also linked to the likelihood of NAb seroconversion failure in PLWH. Our results were similar to those of Zhang et al. (45), who found that healthy vaccine recipients with low lymphocyte counts failed to undergo NAb seroconversion. Our findings also supported the view that an additional vaccine dose may be necessary for PLWH with lower absolute lymphocyte count (46).

We further constructed a risk model to assess the serological status with respect to NAb seroconversion after vaccination. To the best of your knowledge, this is the first report of a model for predicting the outcome of NAb seroconversion in PLWH. We showed that the risk model had good predictive ability (AUC = 0.777) and maybe useful for providing tailored guidance on the vaccination of PLWH. To date, serologic status assessment has been shown to be vital in predicting the risk of SARS-CoV-2 infection (47, 48). Thus, we analyzed the association between the SARS-CoV-2 Omicron infection rates in PLWH and NAb seroconversion within a year after the third vaccination. We detected only a slight drop in the rate of infection in seroconverters (80.00%), compared with non-seroconverters (83.33%). The inability to efficiently mount a NAb response against the Omicron BA.5.2 variant after the third vaccination could explain this phenomenon.

Despite of the comparable rates of infection, we found that seroconverters displayed fewer initial symptoms on infection by the SARS-CoV-2 Omicron variant. Currently, the researcher are concerned about the clinical symptoms of vaccinated individuals with SARS-CoV-2 infection (49, 50). The Kohler et al. found the reduced occurrence of common COVID-19 symptoms (e.g., impaired olfaction/taste, limb/muscle pain, and chills) in vaccinated anti-SARS-CoV-2 seropositive individuals (51). Our study further showed the association between post-vaccination seroconversion and a reduced numbers of initial COVID-19 symptoms. We speculated that the reduction in the number of symptoms meant that individuals who achieved vaccine-associated seroconversion likely developed immunity against SARS-CoV-2 (50). In addition, some studies demonstrated that lower numbers of initial symptoms were associated with a lower risk of symptom persistence and less severe COVID-19 (52). The above evidence suggests that seroconverters were less likely to develop severe COVID-19; therefore, more research should focus on achieving adequate levels of anti-SARS-CoV-2 immunity in non-seroconverters.

The present study had some limitations. First, this was a single‐center study with a small PLWH population. Second, further studies are needed to investigate long‐term cellular immunity in addition to the humoral antibody response. Finally, additional work should focus on the evaluation of the risk of reinfection in vaccinated PLWH.

In conclusion, the third dose of the inactivated COVID‐19 vaccine elicited a better NAb response in PLWH when the interval between the second and third doses was extended, especially for PLWH with a CD4 count < 200 cells/μL. Furthermore, our 6-month follow-up results showed that vaccinated PLWH mounted inadequate neutralizing responses to the Omicron variant. Lastly, the risk model highlighted that a longer interval between vaccinations and a high absolute lymphocyte count could increase the likelihood of post-vaccination NAb seroconversion. Collectively, our data will help optimize vaccination strategies in PLWH and highlight the need for developing more effective vaccines against the Omicron variant.

## Data availability statement

The original contributions presented in the study are included in the article/**Supplementary Material**. Further inquiries can be directed to the corresponding author.

## Ethics statement

The studies involving human participants were reviewed and approved by the Clinical Research Ethics Committee of the Hangzhou Xixi Hospital (202109131211000115379) in accordance with the tenets of the Declaration of Helsinki. The patients/participants provided their written informed consent to participate in this study. Written informed consent was obtained from the individual(s) for the publication of any potentially identifiable images or data included in this article.

## Author contributions

JY and YZ contributed equally to this work. The study design was conducted by JY and YZ. The data analysis and manuscript writing were performed by YW and JL. The other authors mainly participated in data collection. All authors were involved in the interpretation of the results and the statistical analyses. All authors contributed to the article and approved the submitted version.
