# Supplementary material for: Extending the dosing interval of COVID-19 vaccination leads to higher rates of seroconversion in people living with HIV
Source: Front Immunol. 2023 Mar 2;14:1152695. doi: 10.3389/fimmu.2023.1152695 (PMC10017959; doi:10.3389/fimmu.2023.1152695)
Supplement: Supplementary file 1 [file Table_1.docx]

**Table S1.** The dynamic changes in the neutralizing antibody levels and titers among cohort 1 pre-3rd and post-3rd vaccination.

| Cohort 1 | | Time | | | *P* value | | |
| --- | --- | --- | --- | --- | --- | --- | --- |
|  |  | T1: 2nd-3m | T2: 3rd-2m | T3: 3rd-6m | T1 vs T2 | T1 vs T3 | T2 vs T3 |
| NAb  (GMC, 95%CI) | | 9.79  (8.43,11.37) | 55.38  (40.57,75.59) | 21.99  (17.05,28.34) | < 0.0001 | < 0.0001 | < 0.0001 |
| Strain | S1: WT  (GMT, 95%CI) | 3.12  (1.71,5.66) | 38.48  (23.22,63.76) | 13.68  (8.66,21.60) | 0.001 | 0.002 | 0.002 |
|  | S2: Delta  (GMT, 95%CI) | 2.00  (2.00,2.00) | 7.28  (4.07,13.05) | 3.38  (2.03, 5.64) | — | — | 0.013 |
|  | S3: Omicron  (GMT, 95%CI) | 2.00  (2.00,2.00) | 2.42  (1.79,3.27) | 2.00  (2.00,2.00) | — | — | — |
| *P* value | S1 vs S2 | — | 0.002 | < 0.001 |  |  |  |
|  | S1 vs S3 | — | < 0.001 | — |  |  |  |
|  | S2 vs S3 | — | 0.0156 | — |  |  |  |

2nd-3m: 3-month after the second vaccination; 3rd-2m: 2-month after the third vaccination; 3rd-6m: 6-month after the third vaccination; T1-3: time 1-3; S1-3: strain 1-3; GMC: geometric mean concentration; GMT: geometric mean titer; CI: confidence interval.

**Table S2.** The comparison of baseline characteristics of participants between two subgroups in cohort 1.

| Characteristics | PLWH < 200  (n = 19) | PLWH ≥ 200  (n = 44) | *P* value |
| --- | --- | --- | --- |
| Baseline NAb | 7.80 (4.94,11.10) | 9.84 (7.15,14.88) | 0.038 |
| Age | 45.72±17.02 | 38.94±11.27 | 0.124 |
| Sex |  |  |  |
| male | 18 (94.74) | 43 (97.73) | — |
| female | 1 (5.26) | 1 (2.27) |  |
| Sexual transmission route |  |  |  |
| Homosexual/bisexual | 13 (68.42) | 34 (77.27) | 0.665 |
| Heterosexual | 6 (31.58) | 9 (20.45) |  |
| Others | 0 (0.00) | 1 (2.27) |  |
| Marital status |  |  |  |
| Married | 8 (42.11) | 13 (29.55) | 0.284 |
| Unmarried | 11 (57.89) | 26 (59.09) |  |
| Divorced/widowed | 0 (0.00) | 5 (11.36) |  |
| Education |  |  |  |
| High school or lower | 8 (42.11) | 11 (25.00) | 0.376 |
| Junior college | 4 (21.05) | 14 (31.82) |  |
| College or higher | 7 (36.84) | 19 (43.18) |  |
| BMI | 21.98±3.24 | 22.48±3.43 | 0.579 |

PLWH < 200: PLWH with a CD4 count < 200 cells/µL; PLWH ≥ 200: PLWH with a CD4 count ≥ 200 cells/µL; BMI: body mass index.

**Table S3.** The dynamic changes in the neutralizing antibody levels and titers among cohort 2 pre-3rd and post-3rd vaccination.

| Cohort 2 | | Time | | | *P* value | | |
| --- | --- | --- | --- | --- | --- | --- | --- |
|  |  | T1: 2nd-5m | T2: 3rd-1m | T3: 3rd-6m | T1 vs T2 | T1 vs T3 | T2 vs T3 |
| NAb  (GMC, 95%CI) | | 8.21  (7.37, 9.14) | 227.03  (180.6,285.4) | 64.59  (51.34,81.26) | < 0.0001 | < 0.0001 | < 0.0001 |
| Strain | S1: WT  (GMT, 95%CI) | 2.10  (2.00, 2.21) | 66.09  (53.76,81.24) | 14.83  (11.86,18.53) | < 0.0001 | < 0.0001 | < 0.0001 |
|  | S2: Delta  (GMT, 95%CI) | 2.00  (2.00, 2.00) | 10.83  (8.75,13.41) | 3.63  (3.04,4.33) | — | — | < 0.0001 |
|  | S3: Omicron  (GMT, 95%CI) | 2.00  (2.00, 2.00) | 2.60  (2.27, 2.98) | 2.00  (2.00, 2.00) | — | — | — |
| *P* value | S1 vs S2 | — | < 0.0001 | < 0.0001 |  |  |  |
|  | S1 vs S3 | — | < 0.0001 | — |  |  |  |
|  | S2 vs S3 | — | < 0.0001 | — |  |  |  |

2nd-5m: 5-month after the second vaccination; 3rd-1m: 1-month after the third vaccination; 3rd-6m: 6-month after the third vaccination; T1-3: time 1-3; S1-3: strain 1-3; GMC: geometric mean concentration; GMT: geometric mean titer; CI: confidence interval.

**Table S4.** The comparison of baseline characteristics of participants between two subgroups in cohort 2.

| Characteristics | PLWH < 200  (n = 25) | PLWH ≥ 200  (n = 70) | *P* value |
| --- | --- | --- | --- |
| Baseline NAb | 6.40 (4.92,9.17) | 7.92 (6.28,9.86) | 0.034 |
| Age | 39.41±13.82 | 37.43±11.66 | 0.488 |
| Sex |  |  |  |
| male | 24 (96.00) | 68 (97.14) | — |
| female | 1 (4.00) | 2 (2.86) |  |
| Sexual transmission route |  |  |  |
| Homosexual/bisexual | 20 (80.00) | 56 (80.00) | 0.228 |
| Heterosexual | 4 (16.00) | 14 (20.00) |  |
| Others | 1 (4.00) | 0 (0.00) |  |
| Marital status |  |  |  |
| Married | 11 (44.00) | 17 (24.29) | 0.151 |
| Unmarried | 13 (52.00) | 46 (65.71) |  |
| Divorced/widowed | 1 (4.00) | 7 (10.00) |  |
| Education |  |  |  |
| High school or lower | 11 (44.00) | 18 (25.71) | 0.222 |
| Junior college | 5 (20.00) | 16 (22.86) |  |
| College or higher | 9 (36.00) | 36 (51.43) |  |
| BMI | 21.75±2.77 | 21.81±4.34 | 0.947 |

PLWH < 200: PLWH with a CD4 count < 200 cells/µL; PLWH ≥ 200: PLWH with a CD4 count ≥ 200 cells/µL;BMI: body mass index.

**Table S5.** The differences on the neutralizing antibody titers between two subgroups in cohort 2 after the third vaccination.

| Geometric mean titer (GMT) | PLWH < 200  (n = 19) | PLWH ≥ 200  (n = 46) | *P* value |
| --- | --- | --- | --- |
| 1-month |  |  |  |
| WT strain  (GMT, 95%CI) | 66.01  (44.0, 99.04) | 66.12  (51.53, 84.83) | 0.9213 |
| Delta strain  (GMT, 95%CI) | 8.82  (5.53, 14.07) | 11.81  (9.29, 15.01) | 0.2329 |
| Omicron strain  (GMT, 95%CI) | 2.36  (1.94, 2.89) | 2.71  (2.27, 3.23) | 0.4094 |
| 6-month |  |  |  |
| WT strain  (GMT, 95%CI) | 14.17  (8.87, 22.67) | 15.11  (11.65, 19.61) | 0.9094 |
| Delta strain  (GMT, 95%CI) | 3.58  (2.46, 5.20) | 3.65  (2.97, 4.49) | 0.7628 |
| Omicron strain  (GMT, 95%CI) | 2.00  (2.00, 2.00) | 2.00  (2.00, 2.00) | — |

PLWH < 200: PLWH with a CD4 count < 200 cells/µL; PLWH ≥ 200: PLWH with a CD4 count ≥ 200 cells/µL.

**Table S6.** The differences on the neutralizing antibody titers against different SARS-CoV-2 strains between two cohorts at 6 months post-3rd vaccination.

| Geometric mean titer (GMT) | Cohort 1 (n = 11) | Cohort 2 (n = 64) | *P* value |
| --- | --- | --- | --- |
| WT strain  (GMT, 95%CI) | 13.68  (8.66, 21.60) | 14.83  (11.86, 18.53) | 0.6719 |
| Delta strain  (GMT, 95%CI) | 3.38  (2.03, 5.64) | 3.63  (3.04, 4.33) | 0.9063 |
| Omicron strain  (GMT, 95%CI) | 2.00  (2.00, 2.00) | 2.00  (2.00, 2.00) | — |

GMT: geometric mean titer; CI: confidence interval.

**Table S7.** The baseline characteristics of healthy controls and PLWH.

| Characteristics | HC (n = 13) | PLWH (n = 158) |
| --- | --- | --- |
| Age (years) | |  |
| <30 | 1 (7.69) | 41 (25.95) |
| 30-40 | 7 (53.84) | 61 (38.61) |
| 41-50 | 2 (15.39) | 24 (15.19) |
| ≥50 | 3 (15.39) | 32 (11.39) |
| Sex |  |  |
| Male | 6 (46.15) | 153 (96.84) |
| Female | 7 (53.85) | 5 (3.16) |
| BMI | 22.30±1.68 | 22.01±3.74 |

HC: healthy control; PLWH: people living with HIV; BMI: body mass index.

**Table S8.** The differences on the neutralizing antibody levels and titers between healthy controls and PLWH at 6 months post-3rd vaccination.

| Neutralizing Antibody | HC (n = 13) | PLWH (n = 158) | *P* value |
| --- | --- | --- | --- |
| Geometric mean concentrations  (GMC, 95%CI) | 101.90  (52.18, 199.00) | 42.03  (34.80, 50.76) | 0.0056 |
|  | HC (n = 13) | PLWH (n = 75) |  |
| WT strain  (GMT, 95%CI) | 15.94  (9.66, 26.31) | 14.65  (12.02,17.86) | 0.8652 |
| Delta strain  (GMT, 95%CI) | 3.87  (2.27, 6.59) | 3.59  (3.05, 4.23) | 0.4531 |
| Omicron strain  (GMT, 95%CI) | 2.00  (2.00, 2.00) | 2.00  (2.00, 2.00) | — |

GMC: geometric mean concentration; GMT: geometric mean titer; HC: healthy control; PLWH: people living with HIV; CI: confidence interval.

**Table S9.** Univariate cox regression analysis was used to analyze the factors of the outcome of NAb seroconversion among PLWH after the 3rd vaccination.

| Variables | *β* value | SE | Wald χ^2^ | *P* value | HR (95%CI) |
| --- | --- | --- | --- | --- | --- |
| Age (years) |  |  |  |  |  |
| ≥50 vs <50 | 0.721 | 0.261 | 7.627 | 0.006 | 2.057 (1.233,3.433) |
| Sex |  |  |  |  |  |
| female vs male | 0.933 | 0.465 | 4.024 | 0.045 | 2.542 (1.022,6.325) |
| Vaccination interval |  |  |  |  |  |
| 2 vs 1 | -1.092 | 0.234 | 21.716 | < 0.0001 | 0.336 (0.212,0.531) |
| Marital status |  |  | 4.397 | 0.111 |  |
| 2 vs 1 | -0.433 | 0.233 | 3.446 | 0.063 | 0.649 (0.411,1.024) |
| 3 vs 1 | -0.76 | 0.535 | 2.016 | 0.156 | 0.468 (0.164,1.335) |
| Education |  |  | 8.741 | 0.013 |  |
| 2 vs 1 | -0.314 | 0.29 | 1.179 | 0.278 | 0.73  (0.414,1.288) |
| 3 vs 1 | -0.761 | 0.258 | 8.68 | 0.003 | 0.467 (0.282,0.775) |
| BMI | 0.017 | 0.029 | 0.325 | 0.569 | 1.017 (0.96,1.077) |
| Time at initiation of treatment/years |  |  | 4.145 | 0.126 |  |
| 2-5 vs <2 | -0.524 | 0.326 | 2.585 | 0.108 | 0.592 (0.313,1.122) |
| ≥5 vs <2 | 0.005 | 0.282 | 0.000 | 0.985 | 1.005 (0.579,1.746) |
| White cell counts (cells/μL) | -0.107 | 0.071 | 2.256 | 0.133 | 0.899 (0.782,1.033) |
| Neutrophils (%) | 0.018 | 0.012 | 2.068 | 0.150 | 1.018 (0.994,1.043) |
| Neutrophils (cells/μL) | -0.057 | 0.09 | 0.401 | 0.527 | 0.945 (0.792,1.127) |
| Lymphocytes (cells/μL) | -0.523 | 0.207 | 6.387 | 0.011 | 0.593 (0.395,0.889) |
| NLRs | 0.254 | 0.095 | 7.094 | 0.008 | 1.289 (1.069,1.553) |
| Hemoglobin | -0.007 | 0.007 | 1.072 | 0.301 | 0.993 (0.98,1.006) |
| Blood platelets | 0.001 | 0.002 | 0.133 | 0.715 | 1.001 (0.997,1.005) |
| CD4^+^CD25^high^CD127^Low^ Tregs (cells/μL) | -0.02 | 0.012 | 2.581 | 0.108 | 0.981 (0.957,1.004) |
| CD4^+^CD25^high^CD127^Low^ Tregs (%) | -0.072 | 0.067 | 1.154 | 0.283 | 0.931 (0.816,1.061) |
| CD4^+^ T-cells (cells/μL) | -0.001 | 0.001 | 0.755 | 0.385 | 0.999 (0.998,1.001) |
| CD45RA^+^ rTregs (cells/μL) | -0.058 | 0.036 | 2.661 | 0.103 | 0.944 (0.88,1.012) |
| CD45RA^+^ rTregs (%) | -0.211 | 0.142 | 2.21 | 0.137 | 0.809 (0.613,1.07) |
| CD45RO^+^ eTregs (cells/μL) | -0.023 | 0.017 | 1.732 | 0.188 | 0.977 (0.945,1.011) |
| CD45RO^+^ eTregs (%) | -0.023 | 0.066 | 0.122 | 0.727 | 0.977 (0.858,1.112) |
| eTreg/rTreg ratio | 0.005 | 0.01 | 0.275 | 0.600 | 1.005 (0.986,1.024) |

SE: standard error; HR: hazard ratio; BMI: body mass index; NLRs: neutrophil/lymphocyte ratios; Tregs: regulatory T cells; rTregs: resting regulatory T cells; eTregs: effector regulatory T cells.

**Table S10.** The optimal cut-off values of continuous variables in predicting the outcome of NAb seroconversion.

| Variables | Associated criterion | Sensitivity (95% CI) | Specificity (95% CI) |
| --- | --- | --- | --- |
| NLRs | >1.96 | 45.68% (34.6%, 57.1%) | 64.94% (53.2%, 75.5%) |
| Lymphocytes (cells/μL) | ≤1.59 | 43.21% (32.2%, 54.7%) | 77.92% (67.0%, 86.6%) |

NLRs: neutrophil/lymphocyte ratios; CI: confidence interval.

**Table S11.** The predictive values of individual variables and risk score in predicting the outcome of NAb seroconversion.

| Variables | AUC (95%CI) | *P* value | Sensitivity (%) | Specificity (%) |
| --- | --- | --- | --- | --- |
| Vaccination interval | 0.737 (0.661,0.804) | < 0.0001 | 62.96 | 84.42 |
| Lymphocytes  (cells/μL) | 0.606 (0.525,0.682) | 0.0038 | 43.21 | 77.92 |
| Risk score | 0.777 (0.704,0.840) | < 0.0001 | 62.96 | 84.42 |

AUC: area under the receiver operating characteristic (ROC) curve; CI: confidence interval.

**Table S12.** The association between the infection rate of SARS-CoV-2 Omicron variant and NAb seroconversion in PLWH after the 3rd vaccination.

| Outcomes | Seroconverters  (n = 60) | Non-seroconverters  (n = 72) | *P* value |
| --- | --- | --- | --- |
| Infection rate (n, %) | 80.00 (48/60) | 83.33 (60/72) | > 0.05 |
| Non-infection rate (n, %) | 20.00 (12/60) | 16.67 (12/72) |  |

**Table S13.** The comparison of baseline characteristics of participants between two groups with SARS-CoV-2-positive.

| Characteristics | Seroconverters  (n = 48) | Non-seroconverters  (n = 60) | *P* value |
| --- | --- | --- | --- |
| Age (years) | |  |  |
| <30 | 14 (21.17) | 14 (23.33) | 0.750 |
| 30-40 | 22 (45.83) | 26 (43.34) |  |
| 41-50 | 6 (12.50) | 12 (20.00) |  |
| ≥50 | 6 (12.50) | 8 (13.33) |  |
| Sex |  |  |  |
| Male | 48 (100) | 57 (95.00) | — |
| Female | 0 (0.00) | 3 (5.00) |  |
| Sexual transmission route | | |  |
| Homosexual/bisexual | 39 (81.25) | 46 (76.67) | 0.340 |
| Heterosexual | 8 (16.65) | 14 (23.33) |  |
| Others | 1 (2.10) | 0 (0.00) |  |
| Marital status | |  |  |
| Married | 11 (22.92) | 20 (33.33) | 0.513 |
| Unmarried | 35 (72.92) | 38 (63.33) |  |
| Divorced/widowed | 2 (4.16) | 2 (3.34) |  |
| Education |  |  |  |
| High school or lower | 8 (16.67) | 19 (31.67) | 0.139 |
| Junior college | 10 (20.83) | 14 (23.33) |  |
| College or higher | 30 (62.50) | 27 (45.00) |  |
| BMI | 21.68±3.10 | 22.36±3.23 | 0.265 |
| Time at initiation of treatment/years | |  |  |
| <2 | 7 (14.58) | 12 (20.00) | 0.201 |
| 2-5 | 20 (41.67) | 15 (25.00) |  |
| ≥5 | 21 (43.75) | 33 (55.00) |  |
| CD4^+^ T-cell counts (cells/μL) | |  |  |
| <200 | 12 (25.00) | 12 (20.00) | 0.369 |
| 200-350 | 16 (33.33) | 28 (46.67) |  |
| 350-500 | 20 (41.67) | 20 (33.33) |  |

BMI: body mass index

**Table S14.** The symptoms of SARS-CoV-2 Omicron variant infection in seroconverted or non-seroconverted PLWH after the 3rd vaccination.

| Symptoms | Seroconverters  (n = 48) | Non-seroconverters  (n = 60) | *P* value |
| --- | --- | --- | --- |
| Fever (n, %) | 37 (77.08) | 48 (80.00) | 0.625 |
| Cough (n, %) | 37 (77.08) | 48 (80.00) | 0.625 |
| Sore throat (n, %) | 19 (39.58) | 23 (38.33) | 0.955 |
| Muscle aches (n, %) | 24 (50.00) | 28 (46.67) | 0.929 |
| Nasal obstruction (n, %) | 20 (41.67) | 24 (40.00) | 0.964 |
| Running nose (n, %) | 16 (33.33) | 21 (35.00) | 0.838 |
| Headache (n, %) | 12 (25.00) | 24 (40.00) | 0.098 |
| Loss of appetite (n, %) | 8 (16.67) | 12 (20.00) | 0.625 |
| Loss of taste and smell (n, %) | 11 (22.92) | 15 (25.00) | 0.821 |
| Abdominal pain and diarrhea (n, %) | 7 (14.58) | 6 (10.00) | 0.565 |
